# Pylorus Preserving Pancreaticoduodenectomy

**DOI:** 10.4103/1319-3767.61229

**Published:** 2010-04

**Authors:** Mehrdad Nikfarjam

**Affiliations:** Department of Surgery, University of Melbourne, Austin Hospital, Melbourne, Australia

Pylorus preserving pancreaticoduodenectomy (PPPD) was first popularized by Traverso and Longmire in 1978.[1] Proponents of this technique believe that it decreases the incidence of post-gastrectomy complications with overall improvements in long-term gastrointestinal function, when compared to classic pancreaticoduodenectomy (PD). Pylorus preserving pancreaticoduodenectomy was initially advocated as an alternative to PD in the setting of chronic pancreatitis, and later utilized for treatment of peri-ampullary malignancy. Although the technique is widely adopted, pylorus preservation in the setting of cancer, remains controversial.

Several studies and reviews have examined outcomes of PPPD compared to classic PD, particularly relating to delayed gastric empting, oncologic safety, and morbidity and mortality.[2] The major conclusions are that PPPD reduces operating time, results in lower blood loss and reduces the need for blood transfusions, with no overall increases in perioperative morbidity or mortality, tumor recurrence or long-term survival. There is however no overall consensus that PPPD is a better technique than classic PD.

Some argue that PPPD increases postoperative morbidity. Warshaw *et al.* were the first to associate delayed gastric emptying with PPPD.[3] One randomized trial of 33 patients had 43% delayed gastric emptying after PPPD compared to zero cases after classic PD resection (*P* < 0.05).[4] The reverse has however also been shown in a randomized trial of PPPD versus radical PD, including antrectomy and extended lymph node dissection showing a 6% delayed gastric emptying rate compared to 16% (*P* = 0.006).[5] Many series indicate no difference in delayed gastric emptying between PPPD and standard PD.[6,7] Based on reviews and meta-analysis there does appear to be at least a trend towards increased delayed gastric emptying associated with PPPD.

Although the oncologic adequacy of PPPD has been a topic of concern, there has been no study that shows reduced mortality or early tumor recurrence following PPPD compared to PD.[2,8] Classic PD must however be considered the operation of choice in cases where there is tumor involvement of the first part of the duodenum or distal stomach. Some also advocate complete removal of the duodenum in cases of periampullary malignancy associated with hereditary syndromes such as familial polyposis coli. In such cases there is increased risk of malignant transformation within any duodenal remnant, due to a genetic field change throughout the duodenum.

The results of reviews and meta-analysis of studies on PPPD need to be interpreted with caution. A clear superiority of one technique over another has not been demonstrated. Trials so far suffer from relevant clinical heterogeneity, small sample size, and a lack of clearly defined outcome definitions. Reviews such as the one presented in this edition of the journal[9] reinforce the need for well-designed, multicenter, international trials, to clearly determine if there is a difference in mortality, morbidity and long survival between PPPD and classic PD. What can be determined from studies so far, is that there are no obvious clinically relevant differences between the techniques, but PPPD appears somewhat faster and causes less blood loss compared to classic PD.
